# Exploring Public Sentiment on the Repurposing of Ivermectin for COVID-19 Treatment: Cross-Sectional Study Using Twitter Data

**DOI:** 10.2196/50536

**Published:** 2025-03-27

**Authors:** Angga Prawira Kautsar, Rano Kurnia Sinuraya, Jurjen van der Schans, Maarten Jacobus Postma, Auliya A Suwantika

**Affiliations:** 1 Unit of Global Health, Department of Health Sciences University Medical Center Groningen University of Groningen Groningen The Netherlands; 2 Department of Pharmaceutics and Pharmaceutical Technology Faculty of Pharmacy Universitas Padjadjaran Sumedang Indonesia; 3 Center of Excellence in Higher Education for Pharmaceutical Care Innovation Universitas Padjadjaran Bandung Indonesia; 4 Department of Pharmacology and Clinical Pharmacy Faculty of Pharmacy Universitas Padjadjaran Sumedang Indonesia; 5 Department of Economics, Econometrics and Finance Faculty of Economics and Business University of Groningen Groningen Indonesia; 6 Center for Health Technology Assessment Universitas Padjadjaran Universitas Padjadjaran Bandung Indonesia

**Keywords:** COVID-19, ivermectin, sentiment analysis, Twitter, social media, public health, misinformation, geolocation analysis

## Abstract

A sentiment analysis of 5051 Twitter posts from January 2022 found that 53.4% of them expressed positive views on ivermectin as a COVID-19 treatment, 35.6% of them were neutral, and 11% of them were negative, highlighting the polarized public perception and the need for careful interpretation of social media data in health communication.

## Introduction

As the COVID-19 pandemic evolves, the scientific community confronts the limitations of vaccines due to emerging viral mutations that potentially decrease vaccine efficacy [1], thus necessitating a parallel investigation into additional therapeutic agents. Ivermectin, a well-established, safe drug, has emerged as a repurposed drug candidate due to preliminary studies suggesting its antiviral properties against SARS-CoV-2 in vitro [1,2]. Nonetheless, the scientific debate remains vigorous, with discussions on the drug’s appropriate formulation and dosing for potential COVID-19 prophylaxis and treatment [3].

Simultaneously, ivermectin’s role has garnered widespread attention on social media, reflecting the public's quest for alternative treatments. Twitter (now X), a hub for real-time public discourse, has become a fertile ground for divergent views on COVID-19 treatment [4,5]. This sentiment analysis focuses on Twitter discussions about ivermectin, showing public opinion that, while not devoid of misinformation risks, these discussions offer an alternative lens to understand the societal pulse on this contentious topic [6]. By examining the sentiments expressed on Twitter, we aim to add nuance to the ongoing discourse, acknowledging the platform's influence on public perception and its implications for health communication strategies.

## Methods

### Overview

This cross-sectional examined the COVID-19–related sentiments on ivermectin using Twitter data. Primary data were collected from Twitter posts about ivermectin worldwide from January 15 to 22, 2022. We searched posts with the keyword “ivermectin” and retrieved raw data with various variables. Data cleaning involved lowercasing capital letters, eliminating retweet symbols, removing punctuation marks, and other preprocessing steps to ensure data accuracy [6]. People’s sentiments were determined by creating a corpus (body of text) and loading a lexicon dictionary based on the positive and negative words. The sentiment score was calculated with the Bing method using a range of –6 to +6 and considered 3 sentiment types: positive, neutral, and negative [7,8]. The Bing lexicon was chosen for its proven effectiveness and simplicity in extensive dataset analysis. Frequency analysis of single terms (unigrams), word pairs (bigrams), and bigram networks helped identify frequently mentioned terms and explore relationships between words [7,8]. Despite its simplicity, this approach is as robust as more complex methods, as it accurately categorizes sentiments and identifies patterns through unigram and bigram frequency analysis [6]. This computational analysis–focused methodology offers valuable insights into public sentiment, complementing traditional clinical evaluation without extensive statistical validation. Data were mined using Twitter’s limited application programming interface (version 2) and analyzed using RStudio (R Foundation) and relevant packages for visualization.

### Ethical Considerations

There was no direct connection with Twitter users. We removed all individual data, usernames, IDs, and tweets in the manuscript and supporting material. Therefore, ethical approval was not required.

## Results

**Figure 1:**
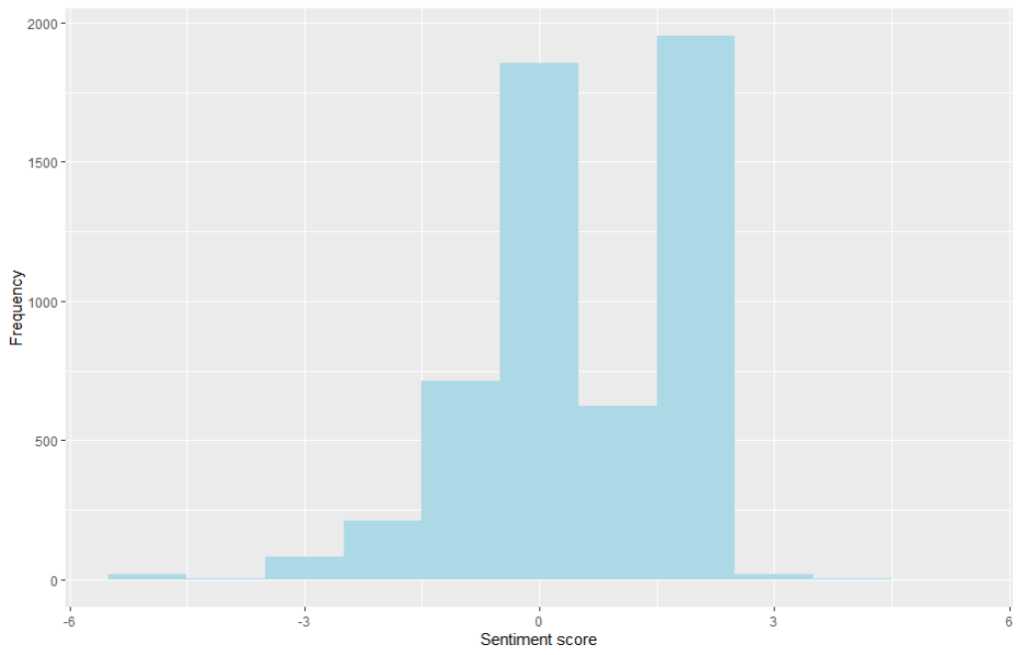
Distribution of sentiment score.

**Figure 2:**
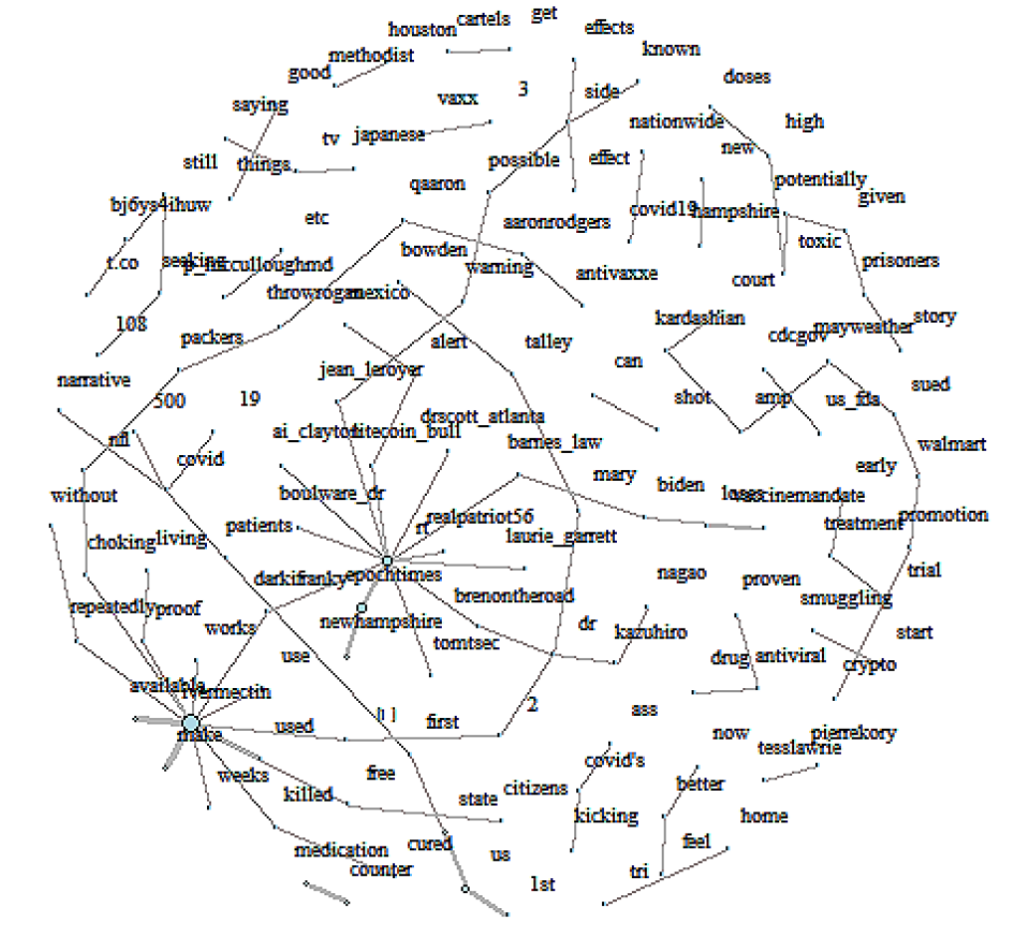
Bigram count network related to tweets ivermectin.

In total, 5051 ivermectin-related tweets underwent sentiment analysis, revealing a prevailing positive sentiment (53.4%), followed by neutral (35.6%) and negative (11%) sentiments among the analyzed tweets (Figure 1). The analysis identified frequently mentioned positive terms such as “medication,” “potentially,” and “treatment,” while negative terms like “poised,” “smuggling,” and “toxic” were less common. Notably, analysis of word pairings unveiled strong associations with positive sentiments, further supporting the notion of ivermectin’s potential efficacy in COVID-19 treatment. Examining these word pairings facilitated in-depth exploration of sentiment patterns within the bigram network, revealing significant connections and relationships in the analyzed tweets (Figure 2).

## Discussion

### Principal Findings

Our analysis shows that tweets about repurposing ivermectin for COVID-19 treatment are predominantly positive. Terms like “medication,” “potentially,” and “treatment” frequently appeared in positive contexts, reinforcing this finding. Phrases such as “ivermectin works,” “available ivermectin,” and “ivermectin medication” were strongly associated with positive sentiment. These results align with previous findings showing ivermectin’s popularity on Twitter for COVID-19 treatment [4,5]. However, some negative sentiment was observed, particularly concerning warnings from the Food and Drug Administration and the limited clinical evidence supporting ivermectin’s efficacy in COVID-19 prevention and treatment [9].

Twitter data have proven valuable in monitoring public responses to the COVID-19 pandemic, as evidenced by a study of millions of SARS-CoV-2–related tweets [10]. This study not only identified dominant topics like new cases, death rates, and preventive measures, but also explored the geographic distribution of sentiments through tweet-embedded geolocation data (code available in Multimedia Appendix 1).

### Limitations

Of note, the findings are based on Twitter data, which may not represent the entire population’s sentiments. Therefore, researchers conducted cross-validation of the sources.

### Conclusions

This sentiment analysis highlights the polarized perception of ivermectin in COVID-19–related discourse and reflects broader public health debates. Given the regulatory advisories and the limited clinical evidence, these public sentiments must be interpreted cautiously.

## Appendix

Multimedia Appendix 1 Code in R.
